# Concentration levels of selected hormones in judokas and the extent of their changes during a special performance test at different ambient temperatures

**DOI:** 10.1186/s13102-023-00751-y

**Published:** 2023-10-23

**Authors:** Tomasz Pałka, Łukasz Rydzik, Łukasz Tota, Piotr Koteja, Tadeusz Ambroży, Dariusz Mucha, Barbara Szpotowicz-Czech, Grzegorz Lech, Norollah Javdaneh, Wojciech Czarny

**Affiliations:** 1 Department of Physiology and Biochemistry, Faculty of Physical Education and Sport, University of Physical Education in Kraków, Kraków, Poland; 2 Institute of Sports Sciences, University of Physical Education, Kraków, Poland; 3 Faculty of Physical Education and Sport, University of Physical Education in Krakow, Krakow, Poland; 4 Academy of Applied Sciences in Nowy Sącz, Faculty of Physical Culture and Security Sciences. Nowy Sącz, Nowy Sącz, Poland; 5 Department of Biomechanics and Sports Injuries, Kharazmi University of Tehran, Tehran, 14911- 15719 Iran; 6 College of Medical Sciences, Institute of Physical Culture Studies, University of Rzeszow, Rzeszów, 35- 959 Poland

**Keywords:** Judokas, Different temperatures, Hormonal Profile

## Abstract

**Background:**

There is little scientific literature available on the diversity of physiological responses of judokas to anaerobic interval exercises in warm environments. Understanding the dynamics of changes in the concentration of selected hormones during a special endurance test at different ambient temperatures may have significant practical value, as it provides an opportunity for optimal programming and monitoring of the training process. So, the main aim of the research was to survey interval anaerobic exercises in different ambient temperatures on Concentration levels of selected hormones in judokas.

**Methods:**

15 judokas athletes (age: 20.65 ± 2.03 years; body height: 178.00 ± 6.31 cm;  body mass: 76.26 ± 12.57 kg; training experience: 12.1 ± 1.57 years) volunteered for the study. The judokas performed five sequences (each lasting 7.20 min) of pulsatile exercises on a cycle ergometer and hand ergometer in a thermoclimatic chamber at temperatures of 21 ± 0.5 °C and 31 ± 0.5 °C. The exercises were different from typical interval exercises, with varying times, upper and lower limb loads, and were followed by a 15-minute break after each sequence. Total duration of the experiment, including the five sequences of pulsating exercise and four 15-minute rest breaks between each exercise sequence, amounted to 96 min and 20 s. The workload was increased by 20 W for the lower limb tests and 12 W for the upper limb tests every 2 min. Biochemical measurements of testosterone (T), cortisol (C), growth hormone (HGH), adrenocorticotropic hormone (ACTH), follicle-stimulating hormone (FSH), adrenaline (E), noradrenaline (NE), and β-endorphin (β-end)were performed using the enzyme-linked immunosorbent assay (ELISA) method on blood samples taken before and after five series of pulsatile exercises, at 1, 24, and 48 h.

**Results:**

Pulsatile exercise at ambient temperatures of 21 and 31 °C resulted in a decrease in body weight of the studied athletes (p < 0.05) and significantly reduced body volume and plasma volume after training (p < 0.05). The concentration of HGH, testosterone, cortisol and NE showed a statistically significant difference after the end of the series of pulsating exercises at both temperatures (p < 0.05) and did not significantly affect the concentration of ACTH, FSH and adrenaline concentration.

**Conclusions:**

An increase in the concentration of growth hormone, cortisol and NE was observed after doing the work at both 21 and 31 °C ambient temperature. Physical exertion in both ambient temperatures contributed to a statistically significant decrease in testosterone concentration. Based on the obtained research results, it can be concluded that physical activity in various thermal conditions of the external environment activates the hormonal response to varying degrees, with the direction of changes depending on the external thermal factor.

**Supplementary Information:**

The online version contains supplementary material available at 10.1186/s13102-023-00751-y.

## Background

Judo is a weight-classified, high-intensity combat sport where the athlete attempts to throw the opponent onto his/her back or to control him/her during groundwork combat [1]. Judo is a complex sport with demands comprising a number of specific characteristics to achieve a high level in competition. It is well known that understanding the characteristics of elite athletes can provide insightful information regarding what is needed for competitive success [1]. Detailed characteristics of the physiological indices of judo competitors have been presented by Franchini et al. [1], who estimated a typical maximal oxygen uptake (VO_2_max) at approx. 50–55 mL^.^kg^− 1.^ min^− 1^. judo are very high intensity sport (blood lactate after fight up to 20 mmol/l) requiring adequate blood buffering capacity that has to be developed in rigorous training. Also, judo involve ample static (armlocks, grappling techniques) and dynamic (throws) muscular work putting higher strength demands on the competitors than do karate or taekwondo [2]. In turn, from the point of view of psychological and hormonal requirements, a direct or indirect contact with the opponent and the risk of injury caused by the fight can be a decisive factor in training and competition requirements [3].The metabolic activity of a judoka during training or tournament fights is based mainly on anaerobic processes [1]. Under these conditions of physical stress, there is a change in hormone levels. The stimulus causing these changes is the mechanical and metabolic stress associated with the physiological cost of exertion, which may be intensified during work in high environmental temperatures [4, 5].The best senior category judo athletes compete in tournaments with 4 to 6 fights. In this competition, the effective time of a single judo fight is 5 min. The time structure of the fight consists of alternating sequences of fighting and breaks. After taking breaks during the fight, its global time is about 460 s. In the case where the competitors do not achieve a point advantage within the limited time of the fight, an additional fight is played, which can last up to another 460 s [6].Considering the fact that numerous international competitions take place under different thermal conditions, which according to some reports may affect the course of physiological and biochemical reactions and the course of the fight, it is worth noting that working in a high environmental temperature activates individual functional systems of the body to a greater extent than exertion in a room temperature environment [7, 8]. The main manifestation of such adaptive reactions is the changes in the heart minute volume [9]. These are conditioned by an increase in the stroke volume (SV) due to the displacement of a larger volume of blood from the blood stores in the abdomen to the skin area and changes in plasma volume (%ΔPV). Intense sweating causes a reduction in the body’s water resources [10]. When the dehydration of the body exceeds 2–3% of body mass, it significantly reduces the ability to perform physical work [10]. This can also lead to the risk of skeletal muscle damage.

Stimulation of the sympathetic nervous system and the hypothalamic-pituitary-adrenal axis are among the main mechanisms of adaptation to physical exercise. The activation results in a mobilization of energy reserves whose extent depends on the type of exercise [11]. It should be pointed out, however, that a similar hormonal response to that caused by physical activity is also evoked by stressful stimuli, including emotions. In combat sports, one finds an overlap of both factors, exercise and fight-related emotions/aggression [12]. Monitoring the endocrine system’s response during training and competitions, particularly peptide hormones (e.g., growth hormone, insulin) and steroid hormones (e.g., cortisol, testosterone), as well as amino acid derivatives (adrenaline, noradrenaline, thyroxine, melatonin, etc.), appears to be essential in training practice. In turn, knowledge of these responses enables athletes to optimize their preparation for competitions. Tropic hormones (adrenocorticotropic hormone and follicle-stimulating hormone) synthesized by the anterior pituitary gland, which model the action of other glands, as well as β-endorphins, are also significant.

Endogenous hormones (testosterone and cortisol) can have a significant impact on cellular metabolism during adaptation to physical exercise and during recovery because they modify anabolic-catabolic processes. Some scientists consider the cortisol-to-testosterone ratio as an indicator of overtraining [13]. The same factors that affect higher levels of ACTH also affect the increase in β-endorphin levels, which, like adrenocorticotropic hormone, is synthesized in the pituitary gland [14]. Exhaustive physical effort can lead to an increase in β-endorphin levels by an average of 200% [15]. However, these changes relate to efforts whose intensity were not lower than 60% VO_2_max and caused an increase in blood lactate levels correlated with the increase in endorphins [16, 17]. Increased secretion of β-endorphin during exercise can reduce the perception of pain and work discomfort, and individuals who engage in regular physical exercise experience satisfaction and faster recovery after exercise [17, 18]. To better understand the action of opioid compounds during physical exercise and after its completion, the profile of β-endorphin changes should be analyzed depending on the duration and intensity of exercise, and compared to the range of changes in other stress hormones [19, 20].

Preparation of professional combat sports athletes for sports competition should include the development of speed-strength and endurance abilities, technical-tactical skills, and volitional traits, as well as the hormonal changes that accompany physical activity [21]. The endocrine system, which plays an important role in maintaining the body’s homeostasis, is a factor that stimulates or inhibits physical work. The sport result also depends on specialized training programs developed by coaching staff based primarily on scientific research in the field of physiology and biochemistry [22]. Such observations make it possible to determine the current level of exercise capacity and also provide the opportunity to monitor physiological and biochemical changes. Sports performance is achieved when all these elements are synchronized and reach an optimal level. During the optimization of the championship level, unfavorable physiological and biochemical changes and overload of the motor system may occur, and even overtraining [23]. The character of judo competitions requires very good psychophysical preparation. It is worth paying attention to the determination of the level of functional and psychological as well as somatic variables, which may affect the conduct of the fight and the final result, through research. Despite the main role of anaerobic processes, a high physical capacity of athletes is necessary to prevent fatigue during training and to rest between successive bouts, as well as for fast and effective recovery after the fight. The analyzed hormonal axis has been shown to be important in the metabolic response to exercise as it is in endurance sports with the dynamic component prevailing [12]. The choice of the research problem seems justified because the effect of anaerobic pulsating physical exercises performed with the upper and lower limbs at different ambient temperatures on changes in physiological and biochemical indicators has been less considered. The results of these studies should enable sports theoreticians and practitioners to optimize training programs. The main aim of the research was to determine whether interval anaerobic physical exercises involving upper and lower limbs in different ambient temperatures would equally affect changes in the levels of selected hormones in judo athletes.

## Methods

### Study design

A group of 15 healthy male professional judo athletes was selected from a pool of 20 candidates, considering their age, training experience, and sports level (each athlete had achieved at least a 5th place ranking in national competitions). Ten athletes completed a full cycle of tests. The experiment received approval from the Bioethics Committee at the Regional Medical Chamber in Krakow (No. 102/KBL/OIL/2011). The study was funded by institutional research funds (7/BS/IFC/2011). The athletes were provided with information about the study’s purpose, methodology, potential risks, and their right to withdraw from the study at any stage without providing a reason, in accordance with the Helsinki Declaration. Written consent was obtained from all participants. The performance tests of the judo athletes were conducted during the competition period at the Department of Physiology and Biochemistry of the University School of Physical Education in Krakow. All tests were performed in the morning hours, at least 2 h after a light meal, considering circadian rhythms. The judo athletes’ tests were divided into two parts: preliminary (stages I and II, as well as III) and principal (stages IV and V). Other the Inclusioncriteria for the research included being above 18 years of age, having good health status, possessing a high sports skill level, having more than 10 years of training experience, and achieving at least a 5th place in national competitions. The exclusion criteria were based on the absence of health issues or discomfort among the subjects. Experiments and the process of conducting the study were conducted under the control of a physician. The sample size was calculated using G*power (v3.1.9.2, Heinrich-Heine-University, Dusseldorf, Germany). With a test power of 80% and a confidence level of 95%, the sample size was estimated to be 15 and was increased to 20 participants for this study.

### Study instruments

During the initial stage of the study, basic biometric measurements such as body height (BH) and body mass (BM) were taken to calculate the Quetelet II index (BMI) following Dubois’ method (Dubois, 1916). Additionally, the lean body mass (LBM) was estimated, and the body surface area (BSA) and body surface area to body mass ratio (BSA · BM^1^) were calculated. Blood pressure (BP) and heart rate (HR) measurements were conducted for diagnostic purposes.

In the preliminary tests of stage II, the participants performed exercise tests to assess the aerobic and anaerobic performance of their lower limbs (LL). After a seven-day interval, the subjects proceeded to stage III, where they underwent the same exercise tests, but this time utilizing their upper limbs (UL). Biometric measurements were taken before conducting the exercise tests in both stages. The Wingate test [20] was employed to evaluate anaerobic performance in both the lower limbs (LL) and upper limbs (UL). Before the main effort, a 5-minute warm-up on a cycle ergometer was performed with an individually adjusted intensity of 50% of VO_2_max. This warm-up included three 5-second maximum accelerations at 2, 4, and 5 min. Two minutes after completing the warm-up, the participants engaged in a 30-second maximum physical effort. In the LL test, the external resistance was set at 8.3% of the subject’s body weight, while in the UL test, it was 4.5% of the body weight [21]. Various indicators from the Wingate test were analyzed during the assessment. Following a minimum two-hour rest period after the Wingate test, the subjects underwent a test to assess their aerobic endurance. This incremental “subjective refusal” test was conducted at the Department of Physiology and Biochemistry, University School of Physical Education in Krakow, at an ambient temperature of 21 ± 0.5 °C and relative humidity of 40 ± 3%. A 2-minute warm-up on a cycle ergometer was performed in both cases, with a pedaling frequency (RPM) of 60 rotations per minute and an intensity of 110 W for LL and 60 W for UL. The workload was increased by 20 W for the lower limb tests and 12 W for the upper limb tests every 2 min. The exercise continued until the participants subjectively felt unable to maintain the desired pedaling rhythm.

In the main study, half of the judo athletes performed five restrictive sequences of pulsating exercise on a leg and arm cycle ergometer within a thermoclimatic chamber at a temperature of 21 ± 0.5 °C (stage IV), while the other half performed the same exercise at a temperature of 31 ± 0.5 °C (stage V), with a relative humidity of 50%±5%. After a seven-day break to eliminate potential residual effects of physical exertion, the subjects repeated stages IV and V, but this time, the first group performed the exercise at a temperature of 31 ± 0.5 °C, while the second group performed it at a temperature of 21 ± 0.5 °C. These exercises deviated from typical pulsating interval exercises in terms of duration, loads applied to the upper and lower limbs, and the inclusion of anaerobic series of physical exertion interspersed with 15-minute rest breaks. A single series of pulsating exercise was conducted according to the schedule provided in Table 1 and repeated five times. The total duration of the experiment, including the five sequences of pulsating exercise and four 15-minute rest breaks between each exercise sequence, amounted to 96 min and 20 s.

**Table 1:** Inclusion and exclusion criteria

20 professional judo competitors	
Included: 15	Excluded: 5
Inclusion criteria	Exclusion criteria
**Age > 18years**	**Age < 18years**
Good health status	Diseases and injuries
High sports skill level	Low sports skill level
Training experience > 10 years	Too short training experience
At least 5th place in national competitions	No success in national competitions
Examination started by 15 participants	
Full scope of tests completed by 10 participants	Full scope of tests not completed by 5 participants

Prior to the pulsatory exercise at both temperatures, a 30-minute acclimation period to the thermal conditions was implemented. This was followed by a 5-minute warm-up, during which individually selected loads equivalent to 50 ± 1% of VO_2_max were utilized. The pulsatory test, conducted separately for the lower limbs (LL) and upper limbs (UL), employed a modified version of the Wingate test. The fundamental parameters of the Wingate test were analyzed during each individual sequence of the pulsatory test. The load applied in each sequence remained consistent and corresponded to 8.3% of the subject’s body mass for LL and 4.5% of the body weight for UL. Blood samples were collected at the beginning and after 5 series of the pulsatory test, as well as at 1, 24, and 48 h, to determine various biochemical and hematological parameters. These included hemoglobin concentration (HGB), hematocrit level (HCT), and changes in plasma volume (%ΔPV). Furthermore, selected hormones were measured in the blood samples, encompassing testosterone (T), cortisol (C), growth hormone (HGH), adrenocorticotropic hormone (ACTH), follicle-stimulating hormone (FSH), adrenaline (E), noradrenaline (NE), and β-endorphin (β-end).

### Measurement technique

The temperature and relative humidity in both the thermoclimatic chamber and the physiological laboratory were continuously monitored using precise instruments. The Harvia thermohygrometer from Finland and the Ellab electronic thermometer from Denmark, with accuracies of ±0.5 °C and ±3% respectively, were employed for this purpose. Air movement was measured utilizing a Hilla catheter thermometer and the simplified Weiss formula, specifically designed for detecting subtle air movements below 1 m · s^-1^. The body height (BH) of male participants was measured with a Martin anthropometer from the USA, accurate to 0.5 cm. Body mass (BM) was measured using a Sartorius F 1505 – DZA electronic scale from Germany, with an accuracy of 1 gram. The measurements of BH and BM were subsequently utilized to calculate the Quetelet II index (BMI), with the adopted norms for the study group ranging from 18.5 to 24.99. Values below 18.5 were classified as underweight, while values above 25 were considered indicative of overweight status. The assessment of percentage body fat (PBF), body fat mass (MBF), and lean body mass (LBM) was conducted using an eight-electrode bioelectrical impedance JAWON MEDICAL IOI-353 body composition analyzer, certified with EC0197 from Korea. The complete blood count (CBC) was performed on the whole blood sample using electro-impedance and photometric analysis with a Analyser HA-22/20 hematological system from CLINDIAG SYSTEMS, Belgium. Blood sampling for biochemical measurements was performed immediately before the pulsatile tests performed by the subjects, one hour after the completion of the exercise tests, and 24 and 48 h after the exercise.

Telemetric recordings of heart rate (HR) during laboratory tests were captured using a Polar 610 S kardiomonitor (Polar Elektro, Finland). The level of dehydration was assessed by measuring body weight (with a precision of 1 g) before and after the exercise sequence, as well as by monitoring urine volume. Respiratory exchange parameters during the graded exercise test were analyzed in 30-second intervals using the computerized Ergospirotest device (Medikro OY, Finland) model M 9427. The lower limb graded exercise test was conducted on a Jeager ER 900 D-72,475 BIT2 cycle ergometer (Germany), while the upper limb graded exercise test was performed on a Monark 891E ergometer (Sweden). The anaerobic (pulsed) tests were conducted in a thermoclimatic chamber at temperatures of 21 and 31 degrees Celsius for the lower and upper limbs, respectively, following a warm-up on a Monark 827E (LL) and 881E (UL) cycle ergometer. The main part of the test was executed on Monark 875E ergometers for the lower limbs and 891E ergometers for the upper limbs.

Blood sampling for hematological analyses, Blood sampling for biochemical analyses, Methodology for the determination of blood indicators using the enzyme-linked immunosorbent assay (ELISA), Calculation of changes in plasma volume, Physiological measurements are explained in detail in the attached file. More details are reported in Appendix 1, available online.

### Statistical methods

The statistical analysis of the numerical data was performed using the Statistica 9.0 for Windows software (StatSoft). Descriptive statistics, including mean and standard deviation (SD), were calculated to summarize the basic characteristics of the data. To assess the changes in parameters resulting from pulsatile exercise, a multivariate analysis of variance was conducted, assuming a normal distribution of the data. The normality of the distributions was evaluated using the Shapiro-Wilk test. In cases where significant statistical differences were observed, the post-hoc Tukey test was employed to assess the strength of these differences.

## Results

### Preliminary results

The demographic characteristics of the subjects are reported in Table 3. Results of the Wingate test for the lower (LL) and upper limbs (UL) and changes in blood biochemistry are presented in Table 2. In the lower limb test, the maximum anaerobic power (RPP) was 12.12 ± 0.87 W^.^kg^− 1^, while in the upper limb test it was 7.00 ± 0.56 W^.^kg^− 1^, which was 42.2% lower. The total work (TW) in the lower limb test was 21.85 ± 4.26 kJ and in the upper limb test it was 13.36 ± 2.50 kJ, resulting in a difference of 38.6%.

**Table 2:** Characteristics of a single series of alternate pulsating efforts

LL	interval	UL	interval	LL	interval	UL	interval	LL	interval	UL	interval	LL	interval	UL	interval
15s.	30s.	15s.	30s.	30s.	60s.	30s.	60s.	20s.	45s.	20s.	45s.	15s.	30s.	15s.	30s.

UL- upper limbs, LL- lower limbs

**Table 3:** The demographic characteristics of the subjects, Index of the Wingate test for the lower (LL) and upper limbs (UL), and Values of physiological indicators recorded in the graded exercise test

Variable	$\bar{x}$ SD	
Age (years)	20.65 ± 2.03	
Height(cm)	178.00 ± 6.31	
body mass (kg)	76.26 ± 12.57	
lean body mass (kg)	64.00 ± 10.42	
body surface area to body mass ratio (cm²·kg^1^	0.0249 ± 0.0016	
VO2max (mL.^.^ kg^− 1^^.^min^− 1^)	43.23 ± 7.79	
anaerobic performance (W·kg^1^)	12.12 ± 0.0001	
Index	LL $\bar{x}$ SD	UL $\bar{x}$ SD
anaerobic performance (W^.^kg^− 1^)	12,12 ± 0,87	7,00 ± 0,56
total work (kJ)	21,85 ± 4,26	13,36 ± 2,50
VO_2_max [mL^.^ kg^− 1^ · min^− 1^]	43,23 ± 7,79	37,19 ± 5,26
HRmax [sk. · min^− 1^]	185 ± 8,19	183 ± 8,43

UL- upper limbs, LL- lower limbs

The mean value of maximal oxygen uptake (VO_2_max), considered as the main indicator of aerobic endurance, reached a value of 43.23 ± 7.79 mL.kg^-1.^min^-1^ in the graded exercise test for lower limbs and 37.19 ± 5.26 mL^.^ kg^− 1 .^ min^− 1^ for upper limbs. The difference was 13.97%. The maximal heart rate (HRmax) recorded in the graded exercise test for lower limbs was on average 185 ± 8.19 beats.min^− 1^, while in the upper limb test it was 183 ± 8.43 beats.min^− 1^ (Table 3).

### Selected physiological indicators recorded before and after pulsatile physical exercise at ambient temperatures of 21 and 31 °C

Pulsatile exercise at two different ambient temperatures resulted in a decrease in body weight of the studied athletes, mainly due to exercise-induced dehydration of the body (Table 3). In order to determine changes in body weight due to physical exertion, athletes did not consume any fluids during the exercise.

As a result of exercise-induced dehydration in LL and UL in temperatures of 21 and 31 °C, a statistically significant reduction in body mass (p < 0.05) was observed. After exercise in 21 °C, BM decreased by 0.980 kg, and in 31 °C by 1.560 kg. There was no statistically significant difference between the BM values recorded before and after the exercise series in temperatures of 21 and 31 °C. The decrease in body mass, caused by dehydration during physical exercise in both temperatures, led to a decrease in plasma volume (∆PV%). These changes were not statistically significant in both the measurements between temperatures of 21 and 31 °C, and between the measurements separately in temperatures of 21 and 31 °C. It is worth noting that the athletes performing physical work in a temperature of 31 °C, where there was a greater combined thermal stimulus of exogenous and endogenous origin, depleted the body’s water resources to a greater extent. In the first hour after the end of the pulsatile exercise in a temperature of 21 °C, the plasma volume loss after the exercise series was − 4.83%, while in a temperature of 31 °C it was at the level of -6.75% (Δ 1.92%) (Table 4).

**Table 4:** Mean and standard deviation of body mass (BM kg) in compared temperatures in individual measurements before and after a series of pulsatile exercises and plasma volume (∆PV%) in compared temperatures in individual measurements

Temperature	Measurement	$\bar{x}$	SD
21^°^C	Before	74,68	11,32
	After	73,70^**#**^	10,99
31^°^C	Before	74,87	11,29
	After	73,31^*^	11,12
21^°^C	II-I	-4,83	4,49
	III-I	0,41	6,79
	IV-I	0,78	6,93
	III-II	6,75	10,52
	IV-II	7,06	9,50
	IV-III	0,61	7,45
31^°^C	II-I	-6,75	5,87
	III-I	0,45	7,52
	IV-I	1,59	6,83
	III-II	7,78	6,91
	IV-II	8,14	8,56
	IV-III	1,86	3,36

# significant differences at p < 0.05 at 21^o^C - differences between measurements

* significant differences at p < 0.05 at 31^o^C - differences between measurements

^ significant differences at p < 0.05 level, between temperatures of 21 and 31 °C, separately for each measurement.

### Changes in selected hormones after pulsating exercise at 21 and 31 °C

**Fig. 1:**
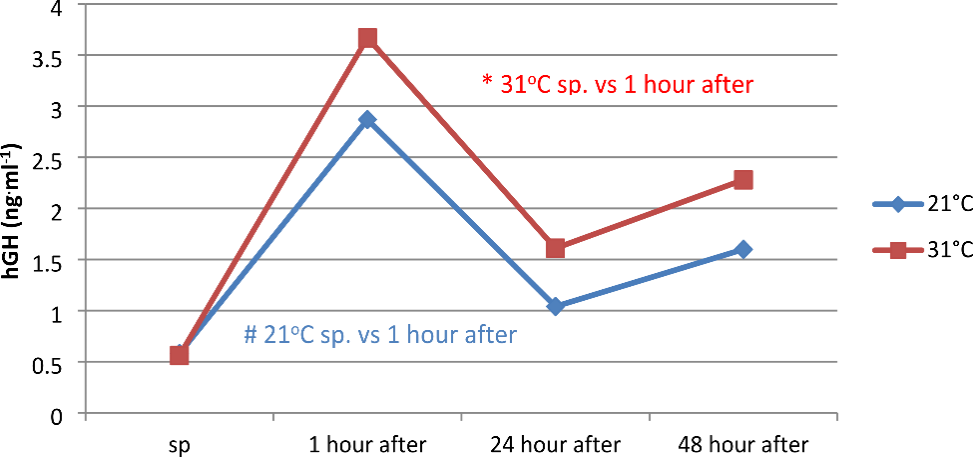
Concentration of growth hormone (hGH) in serum before and at 1, 24, and 48 h after a series of pulsatile exercise at different ambient temperatures

Physical exercise led to significant changes in the concentration of growth hormone (hGH). Assuming significance of differences at the level of p < 0.05, statistically significant differences were found in both temperatures only between resting values and the first hour after the end of physical exercise (Fig. 1). No differences were found in the concentration of this hormone between 21 and 31 °C, separately in individual measurements.

**Fig. 2:**
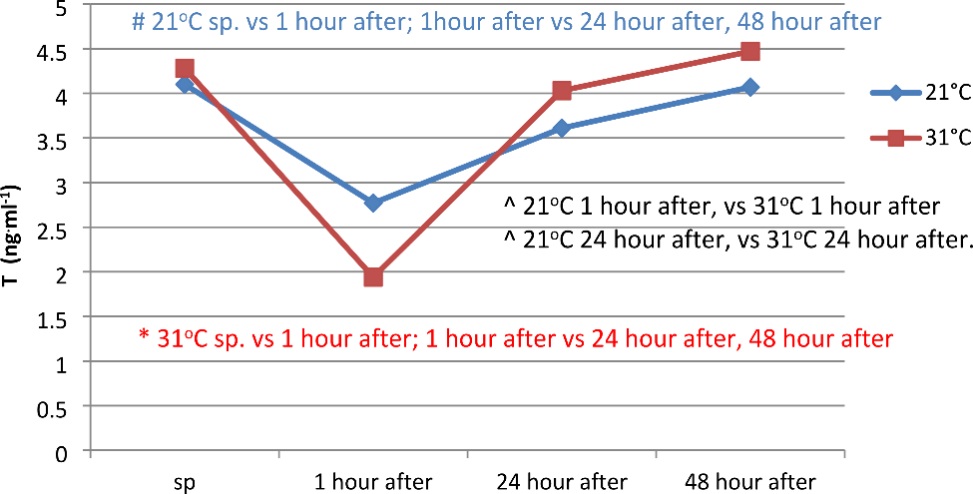
Serum testosterone (T) concentration before and at 1, 24, and 48 h after a series of pulsatile exercises in different ambient temperatures

The concentration of testosterone (T) showed statistically significant differences (p < 0.05) between the resting (sp) measurement and the first hour after the end of the series of pulsatile exercise, as well as between the first hour and 24 and 48 h after the end of the pulsatile exercise sequence at 21 and 31 °C. Differences (p < 0.05) in the level of testosterone concentration were also observed between 21 and 31 °C at 1 and 24 h after physical exercise (Fig. 2).

**Fig. 3:**
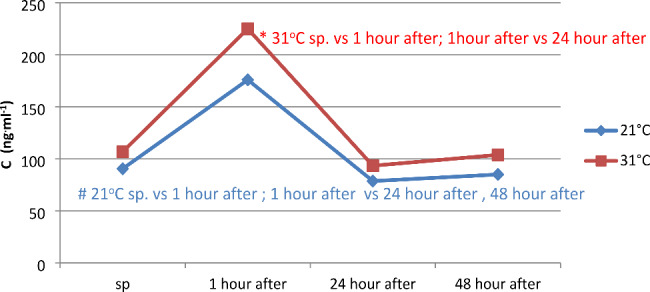
Cortisol (C) concentration in serum before and at 1, 24, and 48 h after a series of pulsatile exercises in different ambient temperatures

There were statistically significant differences (p < 0.05) in serum cortisol (C) concentration between resting (sp) and the first hour after the series of pulsatile exercises, and between the first hour and 24 and 48 h after the completion of the pulsatile exercise sequence at temperatures of 21 and 31 °C. Differences (p < 0.05) in cortisol concentration were also observed between temperatures of 21 and 31 °C at 1 and 24 h after physical activity (Fig. 3).

**Fig. 4:**
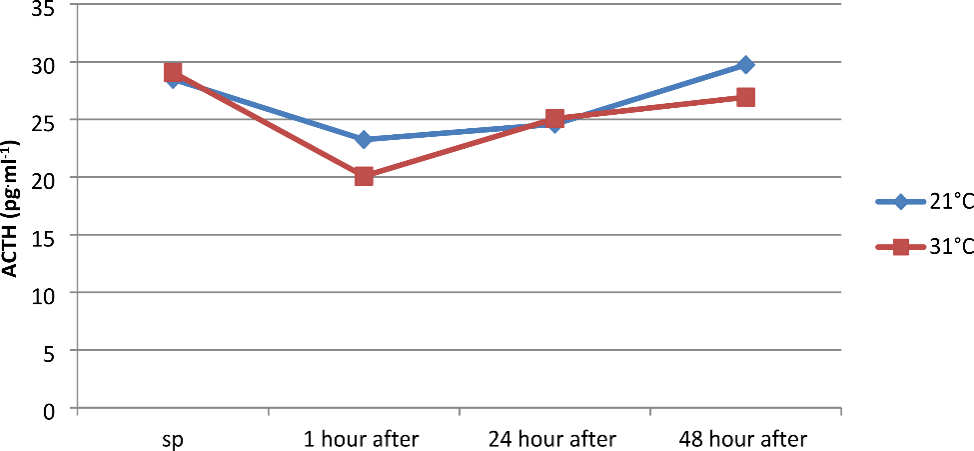
Adrenocorticotropic hormone (ACTH) concentration in serum before and at 1, 24, and 48 h after a series of pulsatile exercise at different ambient temperatures

**Fig. 5:**
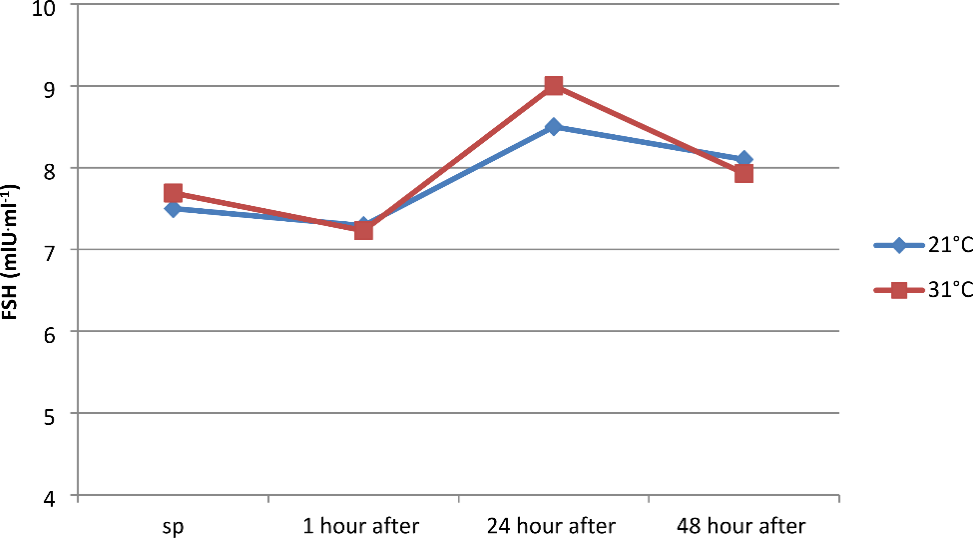
Concentration of follicle-stimulating hormone (FSH) in serum before and at 1, 24, and 48 h after a series of pulsatile exercise in different ambient temperatures

Hormones secreted by the anterior pituitary gland include adrenocorticotropic hormone (ACTH) and follicle-stimulating hormone (FSH). Pulsatile anaerobic exercise series performed at ambient temperatures of 21 and 31 °C did not significantly affect the concentration of ACTH ( pg^.^ml^-1^) and FSH ( mlU^.^ml^-1^) between measurements conducted separately at 21 and 31 °C and between 21 and 31 °C temperatures separately in each measurement (Figs. 4 and 5).

**Fig. 6:**
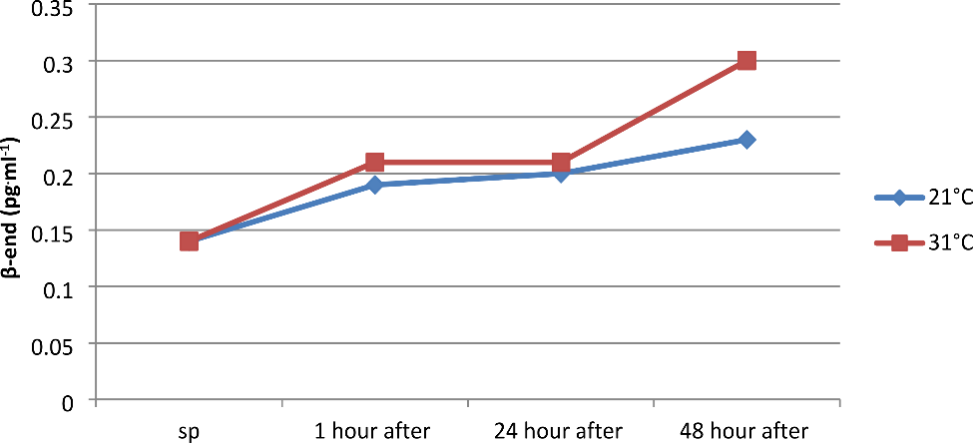
Concentration of β-endorphin in serum before and at 1, 24, and 48 h after a series of pulsatile exercise at different ambient temperatures

Post-exertional changes in beta-endorphin concentration, which are considered a factor reducing pain and stress levels during physical exertion and inducing a sense of well-being after exercise, were statistically insignificant. It is noteworthy that physical exertion performed at a temperature of 31 °C had a slightly greater impact on achieving higher values of β-endorphin concentration (β-end pg^.^ml^-1^), (Fig. 6).

**Fig. 7:**
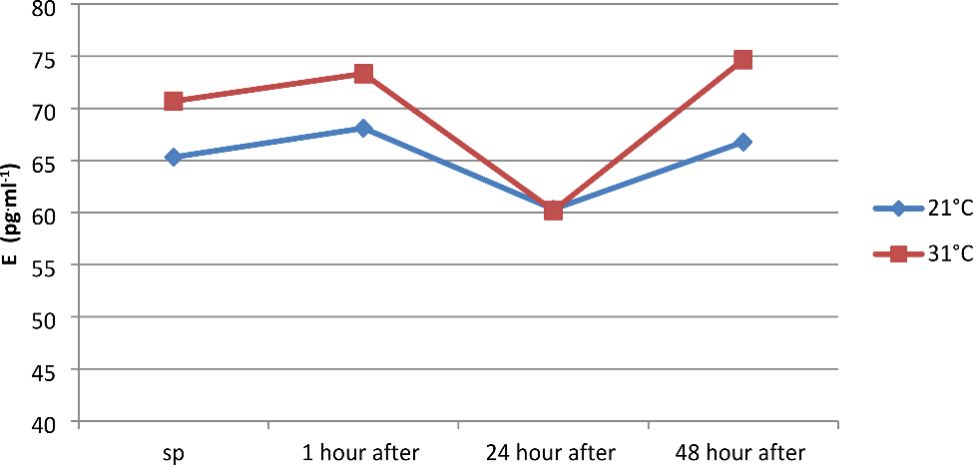
Concentration of adrenaline (E) in plasma before and at 1, 24, and 48 h after a series of pulsatile exercises performed at different ambient temperatures

**Fig. 8:**
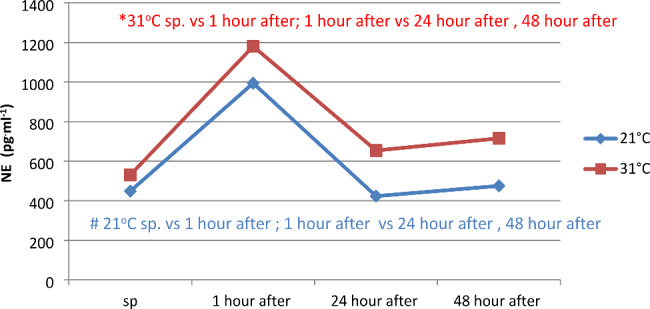
Concentration of noradrenaline (NE) in serum before and at 1, 24, and 48 h after physical exertion in different ambient temperatures

Pulsatile anaerobic exercise sequences performed at ambient temperatures of 21 and 31 °C did not significantly affect changes in adrenaline concentration between measurements conducted separately at temperatures of 21 and 31 °C, as well as between temperatures of 21 and 31 °C separately for each measurement (Fig. 7). Physical exercise at 31 °C had a greater effect on the range of changes in noradrenaline concentration (NE pg^.^ml^-1^), as evidenced by its greater increase (by 650.12  pg^.^ml^-1^), compared to changes registered at 21 °C (545.62  pg^.^ml^-1^) (Fig. 8). Resting noradrenaline concentration was statistically significantly lower than at 1 h after physical exercise at ambient temperatures of 21 and 31 °C (p < 0.05). Statistically significant differences in NE concentrations were observed between measurements at 1 h after exercise and at 24 and 48 h after exercise in both ambient temperatures (p < 0.05). There were no statistically significant differences in post-exercise NE concentration between temperatures of 21 and 31 °C (Fig. 8).

## Discussion

The aim of the observations was to investigate the physiological and biochemical reactions of judokas’ bodies to pulsed anaerobic physical exertion at different ambient temperatures. So far, no scientific information has been found on how much the physiological response of judokas’ bodies to interval anaerobic exertion will vary in warm ambient temperatures. Understanding this knowledge should enable coaching staff to optimize training programs and technologies while maintaining current control of the body’s adaptive changes.The main indicator of anaerobic endurance is the maximal power (RPP) and the total work (TW). The studied athletes had a maximal power of 12.12 ± 0.87 W^.^kg^1^ in the LL test, which was higher than that of Polish judo representatives who had results of 11.46 ± 1.17 W^.^kg^1^ [24], and similar to that of representatives from Canada, Brazil, and Great Britain [1]. In the case of UL, this value was 7.00 ± 0.56 W^.^kg^1^, which was 1.79 W^.^kg^1^ lower than that of the Polish national team players [24]. Representatives from Canada [25] and Brazil [1] as well as Great Britain [26] also had higher maximal power values obtained in the Wingate test performed with the upper limbs. The total work (TW) in J.kg^1^ in the LL test was 285.27 ± 17.73 J.kg^1^ and in the UL test was 173.55 ± 13.50 J.kg^1^. The relative total work (TW J^.^kg^-^^1^) in handball players (245.27 ± 16.1 J^.^kg^-^^1^), football players (251.7 ± 16.0 J^.^kg^-^^1^), and volleyball players (279.9 ± 14.0 J^.^kg^-^^1^) was lower than that of the studied judokas [27]. According to the norms proposed by Zdanowicz and Wojczuk (1984), the studied athletes had a high level of maximal power (RPP) and a very high index of work performed (TW) [27].

The level of judo athletes’ sports performance is primarily determined by their level of anaerobic endurance. However, it should be noted that the level of aerobic endurance is the fundamental factor determining the speed of recovery and is of paramount importance for conducting the second part of the fight [28]. In the studied judo athletes, aerobic endurance measured by the maximum oxygen uptake test performed using the lower limbs were on average 43.23 ± 7.79 mL·kg min^-1^. The maximum oxygen uptake values obtained by the studied athletes differ from the results achieved by world-class athletes, including representatives of Poland, whose values ranged from 56.6 ± 5.6 mL·kg^-1 ^^.^min^− 1^ [29]. Taking into account only the tests performed on the cycle ergometer using the lower limbs, the values for top athletes range from 50 to 61 mL^.^ kg^− 1 .^min^− 1^ [1].

The influence of thermal factors on physical performance is significant both in a positive and negative sense. Dehydration of the body ranging from 2 to 3% reduces physical performance [30]. Among other things, a decrease in endurance, strength, and power of skeletal muscles is observed [30].Observations show that pulsatile exercise series performed at elevated temperatures more strongly strain the bodies of judo athletes than exercises performed at room temperature. Greater thermal stress led to increased activation of sweat glands, as evidenced by changes in body weight and plasma volume. The measure of dehydration after the tests was considered as the decrease in body weight, which was greater in athletes who performed the exercise at a temperature of 31 °C. After exercise at a temperature of 21 °C, body mass decreased by 0.980 kg (1.31%), while at a temperature of 31 °C, it decreased by 1.560 kg (2.08%). Dehydration of the athletes caused by physical exertion and exogenous thermal stimuli led to a decrease in plasma volume. The size of the plasma volume decrease is somewhat indicative of adaptation to physical exertion, especially in unfavorable thermal environmental conditions, and indicates the effectiveness of eliminating heat from the bodies of athletes. It is worth noting that physical training leads to an increase in plasma volume, which may increase the intensity of sweating, eliminating excess heat from metabolic sources, especially during intensive physical activity involving large muscle groups. In the conducted study, the participants performed physical exercise while seated at two different environmental temperatures. Pulsatile exercise series led to insignificant changes in plasma volume. It is worth noting that physical work performed at a temperature of 31 °C led to a greater degradation of plasma water resources by 1.92% compared to exercise performed at a temperature of 21 °C. After 24 h, water resources were close to resting levels.

The stimulating effect of physical exertion on hGH synthesis depends on genetic factors, age, gender, training, and the type of physical exertion. Individuals performing exercise in a warm environment achieve higher hGH concentrations than those performing physical exercises at reduced temperatures. Wideman et al. (2002) indicated that physical exertion, both aerobic and anaerobic, significantly increases the release of hGH [31]. These changes are also conditioned by the load during exercise, the mass of activated muscles, and the breaks between exercises in the case of cyclic activity. Other studies also confirm that the increase in hGH synthesis increases with the intensity of physical exertion [31]. In this research, we found that series of pulsatile exercises caused statistically significant changes in the concentration of growth hormone. Physical exertion performed at an elevated temperature led to an increase in hGH concentration by 555%, and at room temperature by 395%. In the incremental test to exhaustion performed at room temperature and 35 °C, Bridge et al. (2003) showed a similar direction of changes in hGH concentration [32]. This was also confirmed by Vigas et al. (2000) in research conducted on swimmers who performed physical exertion at temperatures of 29 and 35 °C [33].

In the present study, a statistically significant increase in cortisol was observed in judokas after performing physical exertion in both 21 and 31 °C temperatures. Opaszowski et al. (2004) also reported a significant change in cortisol levels in pentathletes in individual events [34]. They observed a decrease of 5.76% after shooting and 17.6% after cross-country running. Swimming competitions caused a 14.7% increase in cortisol levels, while fencing did not induce any changes. The concentration of cortisol is often used as a criterion for adrenal cortex response to maximal physical exertion. It should be noted that post-exercise cortisol concentration can be either positive or negative [35]. Among rowers exercising on a hand cycle ergometer, no changes in cortisol were observed, while tennis players showed slight changes and sprinters showed an increase in cortisol after physical exertion. This response is induced by specific physical exercise, in which the cortisol response to anaerobic exercise is higher than in other athletes [36]. In Cross et al.‘s (1996) study, exercise performed at an elevated temperature led to a threefold increase in cortisol, while exercise performed at room temperature led to a 30% increase, indicating that physical exertion with an exogenous thermal stimulus is a highly stressogenic factor [37]. After the test exercise performed by the studied judokas in an environment at 31 °C, the concentration of cortisol increased by 110.6%, while in 21 °C it increased by 94.35%, which was consistent with the research conducted by Chen (2008) and Parmigiani et al. (2006) [18, 38]. The differences between the changes recorded between the two temperatures were not statistically significant. The concentration of this hormone at 24 and 48 h after exercise was similar to the initial values in both temperatures.

The greater post-exercise cortisol secretion in judo athletes performing physical exertion at 31 °C can be explained by the greater work they performed and the greater increase in Tre. Post-exercise changes in cortisol are dependent on the rate of cortisol uptake from the blood. When the rate of uptake exceeds the rate of release, diverse changes in cortisol levels are observed. Diverse temporal reactions to physical exertion by the adrenal cortex and pituitary gland are a result of delayed cortisol secretion relative to ACTH [39].Cortisol concentration is also considered an indicator of heat stress, and the internal temperature threshold above which its release increases is 38ºC [40]. Taking into account that in studies where pulsatile exercise was performed by judokas in both normal and elevated temperature conditions, the rectal temperature increased above 38ºC in both cases, it can be assumed that the hypothalamic-pituitary-adrenal axis was stimulated, as evidenced by the statistically significant increase in cortisol release into the blood. The greater release of this hormone during exercise in elevated temperatures indicates that it was more stressful work than at room temperature.

In this study, a statistically significant decrease in testosterone concentration was found in the first hour after physical exertion performed at 21 and 31 °C, and these results were consistent with the observations made by Salvador et al. (1987), who registered a decrease in testosterone concentration 45 min after physical exertion. It is noteworthy that physical exertion performed at 31 °C led to a greater decrease in T concentration, and this difference was statistically significant. Parmigiani et al. (2006) observed that after performing the “Kata,“ there was a 4.6% decrease in testosterone concentration, while after “Randori,“ there was a 9% increase [38]. Studies conducted on judo athletes by Chen (2008) also indicate a decrease in T concentration after targeted physical exertion [18]. Brownlee et al. (2005) found a negative correlation between cortisol and testosterone concentration after intense physical exertion, which was not consistent with the observations that showed a positive correlation in the first hour after physical exertion [41]. Lower T values than resting values were observed 24 h after physical exertion, which was consistent with the results obtained for wrestlers [42]. Changes in ACTH concentration depend mainly on the form of physical activity, its intensity and duration, as well as the degree of physical fitness. A factor that increases the synthesis of ACTH is exercise exceeding 80% VO_2_max, which is not consistent with some scientific reports indicating that even physical exercise reaching 80% HRmax may not cause any changes. However, it should be noted that within the first hour after physical exercise at 21 °C, an 18% decrease in ACTH concentration was registered, and at 31 °C, almost 31%.

A statistically insignificant increase in β-endorphin concentration was found, which amounted to 35.7% at 21 °C and 50% at 31 °C. The concentration of this hormone was significantly correlated in the first hour after exercise performed at 21 °C with human growth hormone (hGH) and norepinephrine (NE), while at 31 °C, the concentration of β-endorphin was negatively correlated with epinephrine (E). Cunha et al. (2008) studied individuals participating in marathons and found that post-exercise β-endorphin levels decreased following physical training, which was confirmed in marathon runners. In subsequent marathons, they observed an increase in β-endorphin of 300%, 140%, and 110% in the same individuals. The systematic decrease in β-endorphin levels indicates adaptive changes in the studied athletes. High-intensity physical exertion leads to a series of changes in opioids (peptide hormones), including β-endorphins [43]. The concentration of this hormone depends mainly on the volume and intensity of training, as well as environmental factors and the athlete’s level of sport [19, 44]. However, it should be noted that unlike other hormones, increased synthesis of β-endorphins and adrenocorticotropin occurs only after exceeding 80% VO_2_max, and concentrations recorded in the range of 40–60% VO_2_max are negated by some scientists [19, 44].

However, it should be noted that within the first hour after physical exercise at 21 °C, an 18% decrease in ACTH concentration was registered, and at 31 °C, almost 31%. Scientific studies on changes in FSH levels under the influence of exercise are very scarce, and unfortunately, the few reports available are inconsistent. According to the available information, anaerobic exercises lead to an increase in FSH levels. One theory suggests that this is not due to an increase in secretion but rather a decrease in FSH elimination in the liver. However, to this day, the direction of changes in this hormone, especially in maximal and supramaximal exercises, cannot be unambiguously determined [45]. There is a lack of information based on scientific research regarding the effect of physical training on FSH secretion. In the conducted observations, an insignificantly statistically decrease in the concentration of this hormone was noted, which amounted to 2.8% at a temperature of 21 °C and 5.98% at a temperature of 31 °C. In studies conducted by Chen (2008), a similar direction of changes in this hormone was observed [18]. He found that a 2-hour specialized physical exercise of judo athletes led to a decrease in FSH levels by 28.60%, and its value was 28.63% lower in the first day after its completion compared to the initial level. In the adrenal medulla, catecholamines (E, NE) are synthesized, which function as hormones, and in the nervous system, they act as neurotransmitters. The concentration of these chemical compounds during physical activity depends on the duration and intensity of exercise, as well as the size of the activated muscle groups [46]. Some studies suggest that intensity plays a primary role in the process of increasing E and NE levels, while others indicate that duration is the main factor shaping NE concentration, and psychological stress only enhances the release of E and NE [47].

Despite intensive research into the biochemical changes that occur during physical activity, only in some sports disciplines can biochemical markers be used to assess athletic performance. In combat sports, this is particularly difficult because the acyclic nature of the effort means that tactical and technical actions also contribute to success. However, monitoring biochemical markers during training cycles provides an opportunity to assess adaptive changes induced by targeted physical training, without which high-level competition is extremely difficult. Based on the above information, it can be concluded that physical exertion significantly affects the functioning of the immune and hormonal systems. However, the scope of changes can have different directions and is dependent on external thermal factors.

## Conclusion

The physical exertion in elevated temperature resulted in greater weight loss due to dehydration of the body and changes in plasma volume than at room temperature. An increase in the concentration of growth hormone, cortisol and NE was observed after doing the work at both 21 and 31 °C ambient temperature. Physical exertion in both ambient temperatures contributed to a statistically significant decrease in testosterone concentration. The concentration of ACTH, FSH and adrenaline hormones did not show any significant difference at both 21 and 31 °C ambient temperature. Based on the obtained research results, it can be concluded that physical activity in various thermal conditions of the external environment activates the hormonal response to varying degrees, with the direction of changes depending on the external thermal factor. In order to increase the body’s tolerance to thermal and exercise stress, it is advisable for professional judokas to conduct training in various environmental thermal conditions.

### Electronic supplementary material

Below is the link to the electronic supplementary material.

Supplementary Material 1

## Data Availability

All data have been included in the manuscript content.
