# Supplementary material for: Concentration levels of selected hormones in judokas and the extent of their changes during a special performance test at different ambient temperatures
Source: BMC Sports Sci Med Rehabil. 2023 Oct 23;15:140. doi: 10.1186/s13102-023-00751-y (PMC10594670; doi:10.1186/s13102-023-00751-y)
Supplement: Supplementary file 1 — Supplementary Material 1 [file 13102_2023_751_MOESM1_ESM.docx]

**Blood sampling for hematological analyses**

A volume of 5 ml of blood was collected from the antecubital vein into an EDTA tube for hematological measurements. The complete blood count (CBC) was performed on the whole blood sample using electro-impedance and photometric analysis with a Vet-Analyser HA-22/20 hematological system from CLINDIAG SYSTEMS, Belgium. This analysis included the determination of hematocrit (HCT) and hemoglobin concentration (Hb) levels.

**Blood sampling for biochemical analyses**

Blood sampling for biochemical measurements was performed immediately before the pulsatile tests performed by the subjects, one hour after the completion of the exercise tests, and 24 and 48 hours after the exercise. Blood was drawn from the vein in the elbow crease into two 6-ml tubes: one containing a clotting activator to obtain serum and the other containing EDTA to obtain plasma. After collection, the blood was centrifuged in a laboratory centrifuge (Centrifuge MPW 351R Med. Instruments Polska) at 2000 rpm for 15 minutes. After centrifugation, the serum and plasma were immediately separated from the erythrocyte mass and transferred to prepared tubes for freezing at -70°C (Artico ULF 390 ChRL freezer). Biochemical indicators were analyzed on secured material within two months after the end of the study.

**Methodology for the determination of blood indicators using the enzyme-linked immunosorbent assay (ELISA)**

ELISA (Enzyme Linked Immunosorbent Assay) is a widely utilized immunological and analytical method for detecting specific proteins in biological samples. It involves the use of monoclonal or polyclonal antibodies that are conjugated with suitable enzymes.

In the immunoenzymatic studies using ELISA, the following hormones were analyzed:

Follicle-stimulating hormone - FSH (DRG Instruments GmbH, Germany, test sensitivity: 0.856 mIU/mL, normal range for males: 2-10 mIU/mL).

Testosterone (DRG Instruments GmbH, Germany, test sensitivity: 0.083 ng/mL, normal range for males: 2-6.9 ng/mL).

Cortisol (DRG Instruments GmbH, Germany, test sensitivity: 2.5 ng/mL, normal range: 8-10 am: 50-230 ng/mL; 4:30 pm: 30-150 ng/mL).

Growth hormone (hGH) (DRG Instruments GmbH, Germany, test sensitivity: 0.5 ng/mL, normal range: below 7 ng/mL after overnight rest).

Noradrenaline (LDN Labor Diagnostika Nord GmbH & Co., Germany, test sensitivity: 50 pg/mL, expected values: below 600 pg/mL).

Adrenaline (LDN Labor Diagnostika Nord GmbH & Co., Germany, test sensitivity: 10 pg/mL, expected values: below 100 pg/mL).

Adrenocorticotropic hormone (ACTH) (DRG Instruments GmbH, Germany, test sensitivity: 22 pg/mL, detection range: 5-500 pg/mL, expected values in a group of healthy Americans: 7.0-63 pg/mL).

Beta-endorphins (Phoenix Pharmaceutical, Inc., Germany, test sensitivity: 0.2 ng/mL).

Noradrenaline, adrenaline, β-endorphins, and ACTH were measured in serum, while FSH, testosterone, cortisol, and hGH were measured in blood serum.

Considering the homogeneity of the study group in terms of certain characteristics such as age, gender, and physical activity, reference values were established for certain indicators (ACTH and β-endorphins). These reference groups are characterized by specific ranges of normal values for each indicator.

**Calculation of changes in plasma volume**

Changes in plasma volume (%ΔPV) were calculated using the formula of Dill and Costill (1974), as modified by Harrison et al. ([1](#_ENREF_1), [2](#_ENREF_2)):

%ΔPV = 100{(HBH1/HBH2)·[100 – (HCT2·0.874)] / [100 – (HCT1·0.874)] – 1}

(where HBH1 and HCT1 are the initial values of hemoglobin concentration and hematocrit, and HBH2 and HCT2 are the values of these indicators after exercise).

Post-exercise biochemical indicator concentrations were corrected for changes in plasma volume using the formula of Kraemer and Brown ([3](#_ENREF_3)):

Wsk = (%ΔPV.0.01.Wpo) + Wpo

where:

Wsk - corrected value

Wpo - post-exercise value.

Hematocrit (HCT%) used to calculate changes in plasma volume was determined by the micro-method using a Unipan MPW-212 centrifuge (Poland), and hemoglobin concentration (HBH g.dL-1) in venous blood was measured using the Drabkin method. For this purpose, 5μL of blood was mixed with 20ml of the Drabkin reagent and the result was read on a spectrophotometer (Specol11, Medson, Poland).

**Physiological measurements**

Telemetric recordings of heart rate (HR) during laboratory tests were captured using a Polar 610S kardiomonitor (Polar Elektro, Finland). The level of dehydration was assessed by measuring body weight (with a precision of 1g) before and after the exercise sequence, as well as by monitoring urine volume. Respiratory exchange parameters during the graded exercise test were analyzed in 30-second intervals using the computerized Ergospirotest device (Medikro OY, Finland) model M 9427. The lower limb graded exercise test was conducted on a Jeager ER 900 D-72475 BIT2 cycle ergometer (Germany), while the upper limb graded exercise test was performed on a Monark 891E ergometer (Sweden). The anaerobic (pulsed) tests were conducted in a thermoclimatic chamber at temperatures of 21 and 31 degrees Celsius for the lower and upper limbs, respectively, following a warm-up on a Monark 827E (LL) and 881E (UL) cycle ergometer. The main part of the test was executed on Monark 875E ergometers for the lower limbs and 891E ergometers for the upper limbs.

1. Costill D, Fink W. Plasma volume changes following exercise and thermal dehydration. Journal of applied physiology. 1974;37(4):521-5.

2. Harrison MH. Effects on thermal stress and exercise on blood volume in humans. Physiological reviews. 1985;65(1):149-209.

3. Kraemer WJ, Patton JF, Knuttgen HG, Marchitelli LJ, Cruthirds C, Damokosh A, et al. Hypothalamic-pituitary-adrenal responses to short-duration high-intensity cycle exercise. Journal of Applied Physiology. 1989;66(1):161-6.
